# Retraction Note: Mutagenesis combined with fermentation optimization to enhance gibberellic acid GA3 yield in *Fusarium fujikuroi*

**DOI:** 10.1186/s40643-023-00644-5

**Published:** 2023-04-03

**Authors:** Ya-Wen Li, Cai-Ling Yang, Hui Peng, Zhi-Kui Nie, Tian-Qiong Shi, He Huang

**Affiliations:** 1 School of Food Science and Pharmaceutical Engineering, Nanjing Normal University, 2 Xuelin Road, Qixia District, Nanjing, 210023 People’s Republic of China; 2 College of Biotechnology and Pharmaceutical Engineering, Nanjing Tech University, No. 30 South Puzhu Road, Nanjing, 211816 People’s Republic of China; 3 Jiangxi New Reyphon Biochemical Co., Ltd., Salt and Chemical Industry, Xingan, China

**Retraction: Bioresources and Bioprocessing (2022) 9:106** **https://doi.org/10.1186/s40643-022-00595-3**

The Editor has retracted this article on request from the authors, because the authors stated they did not have the ownership or permission to publish the data reported here.

All authors agree to this retraction.

